# Infectivity of Norovirus GI and GII from Bottled Mineral Water during a Waterborne Outbreak, Spain

**DOI:** 10.3201/eid2601.190778

**Published:** 2020-01

**Authors:** Susana Guix, Cristina Fuentes, Rosa M. Pintó, Albert Blanco, Aurora Sabrià, Eduard Anfruns-Estrada, Virginia Rodríguez Garrido, Manuel Alonso, Rosa Bartolomé, Thais Cornejo, Tomàs Pumarola, Albert Bosch

**Affiliations:** University of Barcelona, Barcelona, Spain (S. Guix, C. Fuentes, R.M. Pintó, A. Blanco, A. Sabrià, E. Anfruns-Estrada, A. Bosch);; Vall d’Hebron University Hospital, Barcelona (V. Rodríguez Garrido, M. Alonso, R. Bartolomé, T. Cornejo, T. Pumarola)

**Keywords:** norovirus, GI, GII, viruses, genotypes, 50% illness dose, dose causing illness, infectivity, secretor status, bottled mineral water, waterborne outbreak, water cooler, real-time quantitative PCR, Spain

## Abstract

During a waterborne outbreak of norovirus in Spain, we estimated 50% illness doses for a group of exposed (secretor) persons to be 556 (95% CI 319–957) genome copies/day for norovirus GI and 2,934 (95% CI 1,683–5,044) genome copies/day for norovirus GII. Use of a propidium monoazide viability assay reduced these values.

Human noroviruses are a major agent of acute gastroenteritis, are distributed worldwide, and affect all age groups (*1*). One of the largest outbreaks of infection with norovirus, caused by consumption of contaminated bottled spring water, occurred in Spain during 2016 and affected >4,100 persons (*2*). Multiple genotypes (GI.2, GII.2, GII.4, and GII.17) were identified among patients, and high levels of norovirus genomes were quantified in contaminated water coolers.

Differences in susceptibility to infections with different genotypes have been described and depend on expression of histoblood group antigens (*3*), for which expression is determined primarily by the *FUT2* gene. Although secretor-negative persons are resistant to several norovirus genotypes (*3*), symptomatic infections in nonsecretors have been documented for GI.3, GII.1, GII.2, GII.3, GII.6, GII.7, GII.4, and GII.17 (*3*). These differences, which are based on host genetic susceptibility, partially hamper development of dose–response models.

Noroviruses are highly infectious, although infectivity might vary between genotypes, and data on their infectivity are still scarce. Reported doses causing infection in 50% of exposed persons (ID_50_), determined from volunteer secretor adults challenged with a GI.1 from a stool specimen, range from 18 (95% CI 1–4,350) (*4**,**5*) to 2,800 (95% CI, 290–25,000) genome equivalents (*6*).

Using human intestinal organoids, Costantini et al. estimated the ID_50_ for stool specimens containing GII as 440–4,000 genome copies/mL (*7*). Because genome to infectious virus ratios might be sample specific, especially in environmental samples, determining norovirus infectivity from common-source outbreaks and naturally contaminated samples is essential. Data reported for oyster-related norovirus outbreaks by Thebault et al. inferred higher infectivity estimates: secretors had an ID_50_ of 7.1 (95% CI 0.73 to >10^6^) virus genomes/oyster consumed and a dose causing illness in 50% of exposed persons of 32 (95% CI 1.32 to >10^6^) virus genomes/oyster consumed for norovirus GI and an ID_50_ of 1.6 (95% CI 0.74 to >10^6^) virus genomes/oyster consumed and a dose causing illness in 50% of exposed persons of 4.86 (1.24 to >10^6^) virus genomes/oyster consumed for norovirus GII (*8*).

In this study, we estimated the 50% illness dose in conditions of natural exposure to contaminated water. These persons were selected from a group of exposed persons during a large waterborne outbreak in Spain in 2016 (*2*).

## The Study

This study was conducted in accordance with the Declaration of Helsinki, and approved by the Ethics Committee of the Hospital Universitari Vall d’Hebron (PR[AG]211/2016). Informed written consent was obtained from all persons.

We provided a questionnaire on water consumption, occurrence, type and duration of gastroenteritis symptoms, and blood type (ABO) to 26 persons who had been exposed to drinking water from a water cooler in Spain during 2016. Acute gastroenteritis was defined as vomiting or diarrhea (>3 loose stools within 24 hours) and >2 of the following: nausea, abdominal pain, or fever (temperature >37.8°C). We collected saliva samples to determine secretor status by genotyping the *FUT2* gene (*9*). We collected stool specimens during the symptomatic phase from 13 of 15 symptomatic patients and screened for norovirus by real-time quantitative PCR as described (*10*). We also performed genotype analysis for polymerase and capsid genes (*11*) and used probit analysis to determine the 50% illness dose.

A total of 69% of persons were secretors and 31% were nonsecretors (Table 1). The overall attack rate for symptomatic infection was 58%: 67% for secretors and 38% for nonsecretors. Percentages of secretors were 80% for symptomatic persons and 55% for asymptomatic persons. According to water intake, 62% reported consuming 200–600 mL/day, 23% reported consuming 601–1,000 mL/day, and 15% reported consuming >1,000 mL/day. We found no major differences in water consumption between secretors and nonsecretors. An increase in the proportion of symptomatic persons and the amount of water consumed daily was observed only for secretors (Figure). Gastroenteritis developed in 3 of 8 nonsecretors even though they had not ingested the largest amount of water.

**Table 1:** Characteristics of 26 exposed persons during a waterborne outbreak of norovirus, Spain*

Person no.	Age, y	Duration of symptoms, days	Daily water intake, mL†	Secretor status	ABO blood type	Virus genogroup	Virus genotype
1	46	2	200–600	+	NA	I	GI.P2_GI.2
2	25	2	200–600	+	NA	II	GII.P17_GII.17
3	58	4	200–600	+	NA	II	GII.P17_GII.17
4	45	5	200–600	–	O	I	GI.P2_GI.2
5‡	29	3	200–600	+	O	NA	NA
6‡	31	1	200–600	+	O	NA	NA
7	34	2	200–600	–	O	I	GIP2_GI.2
8	42	4	200–600	+	O	I + II	GI.P2_GI.2
9	45	5	601–1,000	–	NA	I	NA
10	32	3	601–1,000	+	A	I + II	GI.P2_GI.2 and GII.P17_GII.17
11	29	4	601–1,000	+	B	I + II	GII.P17_GII.17
12	26	6	601–1,000	+	O	I + II	GI.P2_GI.2
13	41	9	601–1000	+	O	I + II	NA
14	25	4	>1,000	+	NA	I + II	GII.P2_GII.2
15	55	2	>1,000	+	A	I + II	GI.P2_GI.2
16‡	27	0	200–600	–	NA	NA	NA
17‡	52	0	200–600	–	AB	NA	NA
18‡	54	0	200–600	+	NA	NA	NA
19‡	47	0	200–600	+	O	NA	NA
20‡	59	0	200–600	+	O	NA	NA
21‡	30	0	200–600	+	O	NA	NA
22‡	59	0	200–600	–	O	NA	NA
23‡	NA	0	200–600	+	O	NA	NA
24‡	33	0	601–1,000	+	NA	NA	NA
25‡	38	0	>1,000	–	NA	NA	NA
26‡	42	0	>1,000	–	NA	NA	NA

*NA, not available; –, negative; +, positive. †Plastic glasses used by persons to drink water had a volume of 200 mL. ‡These persons did not provide a stool sample.

**Figure:**
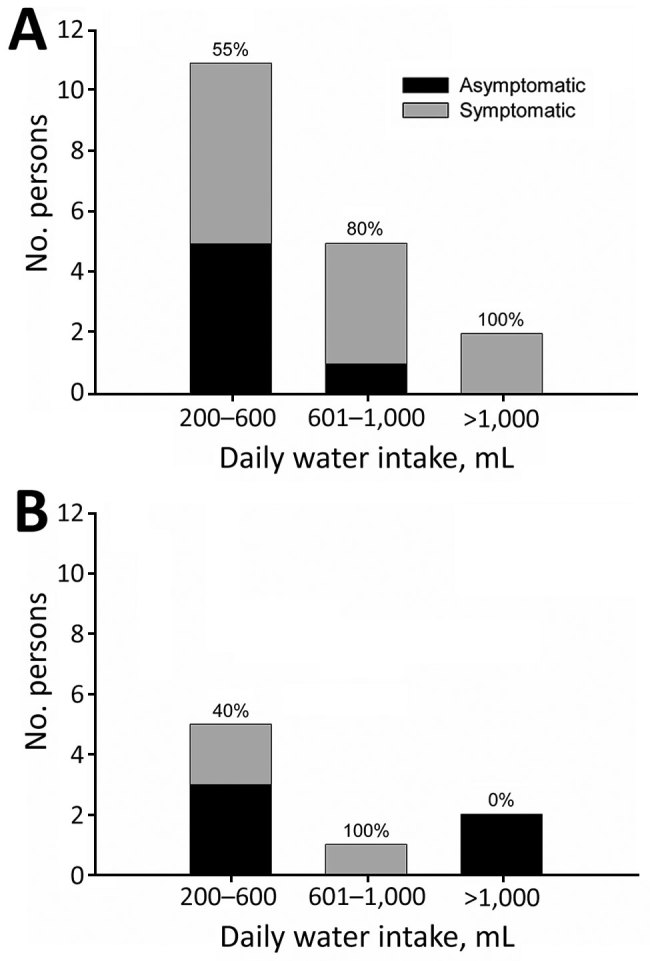
Distribution of A) secretor and B) nonsecretor persons by level of daily water intake and the occurrence of symptoms during a waterborne outbreak of norovirus, Spain. Numbers at the top of each bar indicate percentages of symptomatic persons.

Of 8 exposed nonsecretor persons, 3 (38%) showed development of acute gastroenteritis and only GI viruses were detected in their stool. Although we could not completely rule out that GII viruses might have also infected these persons but were not detected, it is plausible that gastroenteritis in these persons was related to the GI infection. Although binding of GI.2 to nonsecretor histoblood group antigens has been demonstrated in vitro (*12**–**14*), we report clinical infections in nonsecretors. The observed attack rate for a symptomatic GI infection was similar between secretors (50%) and nonsecretors (38%).

We used data for quantification of norovirus in the contaminated water cooler to which the subjects were exposed (*2*) to estimate the average 50% illness dose. High levels of genome copies per liter of water for GI (1.1 × 10^3^) and GII (5.8 × 10^3^) had been detected. Intact virions, estimated by using a viability real-time quantitative PCR, represented <4.4% to 5.6% of the total number of genomes. We estimated 50% illness doses according to the percentages of persons with gastroenteritis and the median water volumes recorded for each group (Table 2). We calculated 95% CIs by using the scenarios corresponding to the minimum and maximum water volumes from each group. The 50% illness dose estimates for secretors were 556 (95% CI, 319–957) genome copies/day for GI exposures and 2,934 (95% CI, 1,683–5,044) genome copies/day for GII exposures. For nonsecretors, the number of infected persons was too low to calculate 50% illness doses.

**Table 2:** Illness status observed for secretors and nonsecretors of norovirus, according to different daily doses of ingested virus genomes, Spain*

Virus genotype	Ingested genome copies/d	Ingested infectious genome copies/d	Secretors			Nonsecretors	
			No. exposed persons	% Symptomatic persons		No. exposed persons	% Symptomatic persons
GI	220–660	10–29	9	22.2		5	40.0
	661–1,100	30–49	5	80.0		1	100.0
	>1,100	>49	2	100.0		2	0.0
GII	1,160–3,480	65–196	9	33.3		5	0.0
	3,481–5,800	197–327	5	80.0		1	0.0
	>5,800	>327	2	100.0		2	0.0

*n = 13. Persons who did not provide a stool sample were excluded from the analysis.

## Conclusions

Our data showed differences in infectivity between norovirus GI and GII, which differs from what was previously observed in oyster-related outbreaks (*8*). Because the number of persons studied was low in both studies, factors that might modify how a particular person responds to virus exposure might partially explain these different observations. In addition, factors related to particular genotypes involved in the outbreaks might also influence infectivity. Although values estimated in this study fall within the range of ID_50_ and 50% illness dose data obtained by other investigators (*4**,**6**,**8*), we observed that median values extrapolated from oyster-related outbreaks (*8*) are the lowest values. This trend might be caused by natural variability, different preimmune status of exposed persons, or strain differences, or the proportion of noninfectious genomes with respect to total genome copies might be lower within contaminated oysters.

Because of the interaction of norovirus with specific carbohydrates found in oyster intestinal tissues (*15*), these animals might specifically bioaccumulate intact infectious virions. In this regard, in our study, after we considered only those genomes incorporated in undamaged capsids, resulting 50% illness doses for secretors were only 25 (95% CI 15–42) for GI and 165 (95% CI 95–284) for GII.

Because the proportion of infectious genomes might vary in each particular environmental scenario, infectivity data for norovirus relating to total and infectious genomes should be consistently investigated whenever possible for a better refinement of risk assessment approaches. Outbreaks caused by consumption of contaminated water and shellfish are often caused by multiple virus strains. Although this factor might hamper infectivity estimates, these types of studies are nevertheless valuable to risk managers and regulators for future formulation of guidelines on acceptance levels for norovirus in specific matrices.
